# Simulating aortic root replacement for a surgical wet laboratory in a resource-limited setting: an economical innovation

**DOI:** 10.1093/icvts/ivad167

**Published:** 2023-10-16

**Authors:** Mian Mustafa Kamal, Abdul Ahad Sohail, Neelam Jawed Qureshi, Haseeb Ullah, Syed Shahabuddin, Mian Yasir Kamal

**Affiliations:** Department of Cardiothoracic Surgery, Aga Khan University Hospital, Stadium Road, Karachi, Pakistan; Department of Cardiothoracic Surgery, Aga Khan University Hospital, Stadium Road, Karachi, Pakistan; Department of Cardiothoracic Surgery, Aga Khan University Hospital, Stadium Road, Karachi, Pakistan; Rehman Medical College, Hayatabad, Peshawar, Khyber Pukhtoonkhwa, Pakistan; Department of Cardiothoracic Surgery, Aga Khan University Hospital, Stadium Road, Karachi, Pakistan; Department of Cardiology, Khyber Teaching Hospital, University Road, Peshawar, Khyber Pukhtoonkhwa, Pakistan

## Abstract

Traditional cardiac surgery residency programs rely mainly on teaching surgical skills in the operating room. The increasing complexity of cardiac surgical operations on high-risk patients and the time constraints placed on residents in this surgical discipline negatively impact the learning opportunities for those residents. Simulation models, though efficient, are very expensive. In Third World Countries, they are unavailable for trainees due to financial constraints.

We have introduced an innovative and cost-effective way of simulating aortic root replacement in a wet laboratory by applying a hand-made valve conduit or ‘pencil conduit’ to a bovine heart. It is reproducible, easy to assemble, cost-effective and simple to use. It can help develop and enhance the surgical skills of residents and junior surgeons for this advanced operation, which requires a meticulous surgical technique performed within a limited time frame.

## MANUSCRIPT

The ultimate goal of a surgical residency programme is to train its residents to be fully competent to perform the surgical skills required to work as independent surgeons. Traditional cardiac surgery residency programmes rely mainly on teaching in the operating room (OR) to improve the residents’ cognitive and technical skills. The learning strategy based on the famous surgical dictum “see one, do one and teach one” may not be applicable in cardiac surgery training for a range of reasons including the complexity of the operations, high-risk patients, time constraints dictated by the use of cardiopulmonary bypass and cross-clamping, concerns about surgical outcomes and ethical issues (1–3). Limitations in the working hours of residents, decreases in patient volume and increases in the numbers of trainees per unit have also negatively affected the learning opportunities in the OR (3,4).

Some procedures like aortic root replacement are too complex and advanced to be taught to residents only in the OR. However, learning these complex procedures is crucial for a junior surgeon. To overcome these hurdles, simulation has made headway in surgical training programmes including those in cardiac surgery (2, 3). In one study, Fan *et al.* showed that after 1 week of practice on a coronary anastomosis simulation model, residents exhibited a 20% decrease in task completion time (5).

Simulation models, though efficient, are expensive. Due to financial constraints, simulation models in Third World Countries are a luxury that most training institutes are unable to afford (2, 6).

To help cardiac surgery residents learn aortic root replacement skills, we have introduced a simple, cost-effective method for simulating aortic root replacement in a wet laboratory. It will help residents simulate the operation and practice the procedure repeatedly outside the OR until they are confident enough to reproduce it in the OR.

### Prerequisites

**Figure 1:**
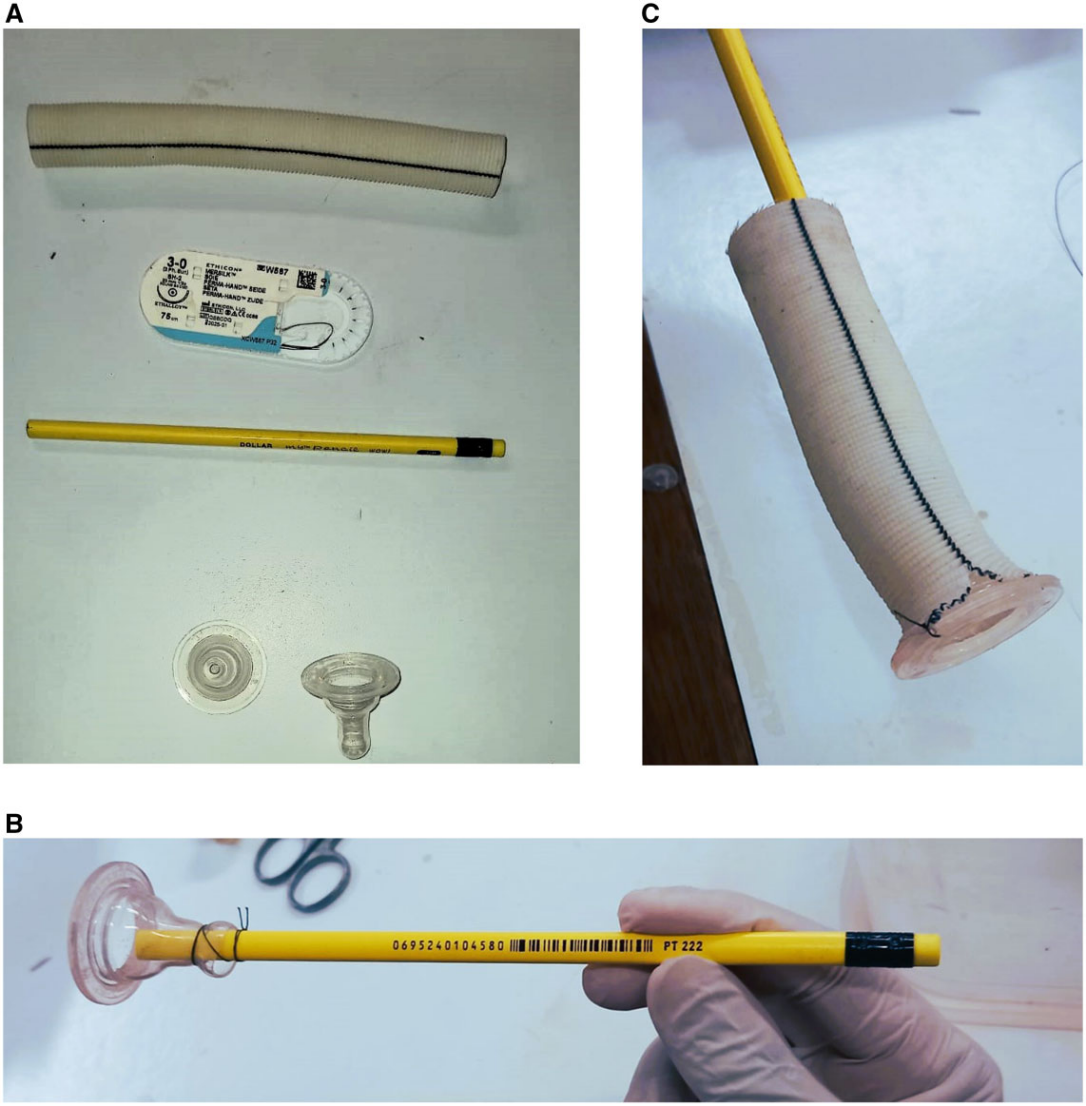
(**A**) Prerequisites for hand-made valve conduit (pencil conduit). (**B**) Pencil introduced in the cut end of the nipple and tied with a silk suture/tie. (**C**) Pacifier and pencil introduced in the Dacron tube graft. Slit made to adjust the size.

This simulation model requires the following set-up:

1. Bovine heart (1 USD)
2. Plastic container box (0.5 USD)
3. Hand-made valve conduit (pencil conduit) (Fig. 1) (0.5 USD)
  - Pencil/wooden rod
  - Pacifier
  - Silk suture (any size 2/0, 3/0) and silk threads
  - Dacron tube grafts (leftover tube grafts from the OR that were not used for an operation)
4. Resident’s surgical instrument set
5. Leftover trocars of chest tubes
6. Sutures

### Steps for making a pencil conduit

**Figure 2:**
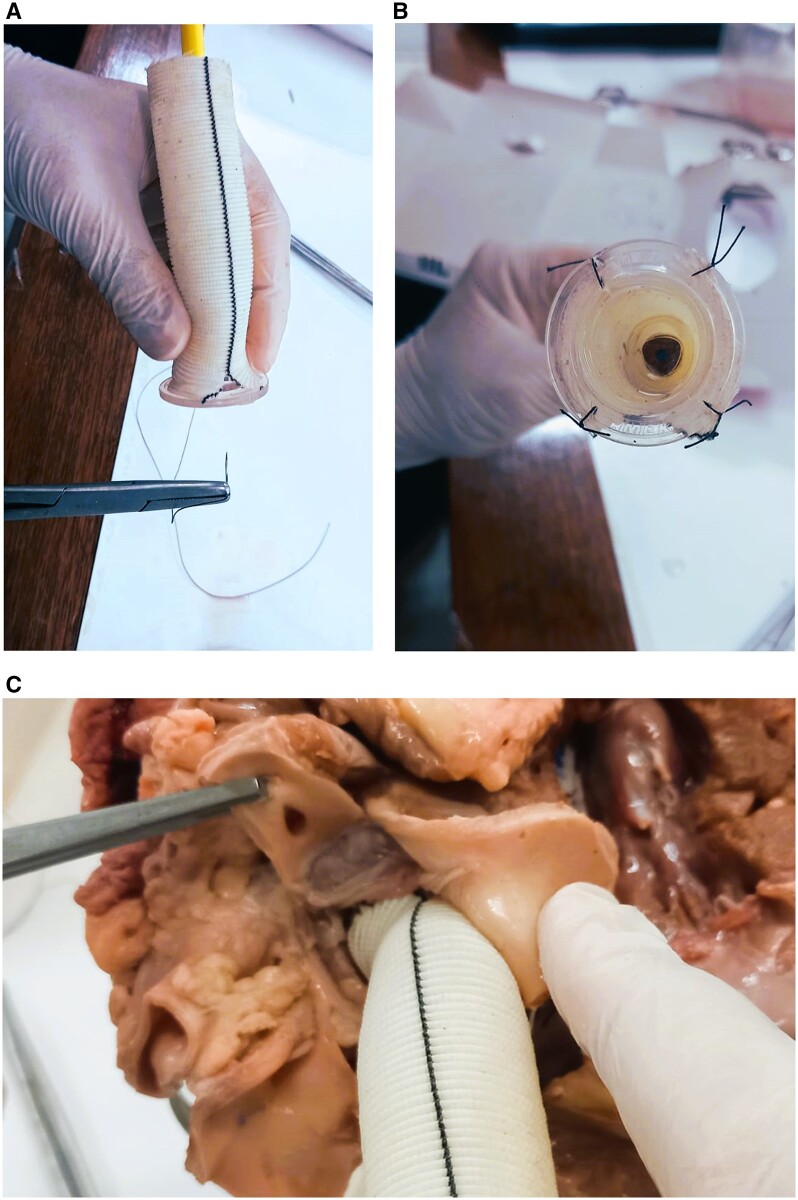
(A) Pacifier sewn to the Dacron tube graft. **(B)** Final look of the hand-made valve conduit (pencil conduit)—inferior view. **(C)** Right coronary button of a bovine heart being prepared to be anastomosed to the Dacron graft conduit. A buttonhole needs to be made on the Dacron tube before completing the anastomosis.

To mimic a real valve conduit for root replacement, we have designed a pencil conduit that is made using a left-over Dacron tube graft from a previous operation, a regular pencil, a pacifier and a silk suture 2/0 (Fig. 1A):

1. The nipple is removed from the pacifier. A small hole is made at the end of the nipple through which the pencil can pass.
2. The 2B pencil is introduced into the cut end of the nipple and tied with a silk thread that will hold the pencil in the nipple (Fig. 1B).
3. The pacifier and the pencil are then introduced into the Dacron tube graft. To adjust the size of the Dacron tube graft to the pacifier, a small slit can be made at the opposite ends of the circumference of the tube graft (Fig. 1C).
4. The outer margin of the pacifier is sewn to the margin of the Dacron tube graft using interrupted/continuous 2/0 silk suture (Fig. 2A).
5. The final pencil conduit, which is similar to a real valve conduit for aortic root replacement, is shown in Fig. 2B.
6. The pencil acts as a handle for the first assistant to stabilize the pencil conduit.
7. Once the conduit is sewn proximally at the aortic valve annulus in the bovine heart, a scalpel can be used to cut the thread at the end of the nipple, which was tied initially to hold the pencil in the nipple. The pencil can then be removed, and the Dacron tube graft can be sewn distally to the remaining ascending aorta.

### Performance of the operation

At the Aga Khan University Hospital, Karachi, Pakistan, we have started the Aortic Root Replacement Wet Laboratory for residents. A brief lecture on the surgical anatomy of the aortic root is given by one of the facilitators. It is followed by a live demonstration for all residents of the procedure by the attending cardiac surgeon. The bovine heart is placed in a plastic container in the desired anatomical position and stabilized using a pair of used trocars. The ascending aorta is transected just above the sinotubular junction, and the anatomy of the root is inspected. The aortic root is excised, and the coronary buttons are harvested. The pencil conduit is then brought into the surgical field to be anastomosed with the aortic annulus. The pencil is then removed from the pacifier by dividing the silk suture. The coronary buttons are then anastomosed on the tube graft after the creation of buttonholes at appropriate positions, as is done in the OR (Fig. 2C).

The residents are then divided into 3 to 4 groups, with each group having 2 residents at separate stations and supervised by a facilitator. Each resident is observed and given feedback at the same time. The whole set-up requires about 600 Pakistani Rupees (approximately only 2 US dollars).

Wet laboratories are a cost-effective way of teaching surgical skills (7). The main limitation for practicing cardiac surgical procedures in a wet laboratory is the unavailability of prostheses (6). The simulation model that we have presented is cost effective, simple and quickly assembled. It allows cardiac surgical residents and junior faculty members to receive training in a resource-limited setting to practice such an advanced procedure safely. It may improve their operating time, visual assessment and correct suture placement using microvascular instruments. They can reproduce it in the OR in elective and emergency procedures and may improve patient outcomes. We need to conduct multiple wet laboratory training sessions on aortic root replacement before we can evaluate whether this method has improved the skills of residents in the OR.

## COMPETING INTERESTS

The authors have declared that no competing interests exist.

## Data Availability

All relevant data are within the manuscript and its supporting information files.
